# Tissue transglutaminase mediates the pro-malignant effects of oncostatin M receptor over-expression in cervical squamous cell carcinoma

**DOI:** 10.1002/path.4222

**Published:** 2013-09-10

**Authors:** Maria M Caffarel, Anasuya Chattopadhyay, Angela M Araujo, Julien Bauer, Cinzia G Scarpini, Nicholas Coleman

**Affiliations:** Department of Pathology, University of CambridgeUK

**Keywords:** tissue transglutaminase, oncostatin M receptor, squamous cell carcinoma, cell migration, invasion, integrin–*α*5*β*1, fibronectin

## Abstract

Oncostatin M receptor (OSMR) is commonly over-expressed in advanced cervical squamous cell carcinoma (SCC), producing a significantly worse clinical outcome. Cervical SCC cells that over-express OSMR show enhanced responsiveness to the major ligand OSM, which induces multiple pro-malignant effects, including increased cell migration and invasiveness. Here, we show that tissue transglutaminase (TGM2) is an important mediator of the ligand-dependent phenotypic effects of OSMR over-expression in SCC cells. TGM2 expression correlated with disease progression and with OSMR levels in clinical samples of cervical and oral SCC. TGM2 depletion in cervical SCC cells abrogated OSM-induced migration on fibronectin-coated surfaces and invasiveness through extracellular matrix, while ectopic expression of TGM2 increased cell motility and invasiveness. Confocal microscopy and co-immunoprecipitation assays showed that TGM2 interacted with integrin–*α*5*β*1 in the presence of fibronectin in cervical SCC cells, with OSM treatment strengthening the interaction. Importantly, integrin–*α*5*β*1 and fibronectin were also over-expressed in cervical and oral SCC, where levels correlated with those of OSMR and TGM2. This combined tissue and *in vitro* study demonstrates for the first time that stimulation of over-expressed OSMR in cervical SCC cells activates TGM2/integrin-α5β1 interactions and induces pro-malignant changes. We conclude that an OSMR/TGM2/integrin-α5β1/fibronectin pathway is of biological significance in cervical SCC and a candidate for therapeutic targeting. Copyright © 2013 Pathological Society of Great Britain and Ireland.

© 2013 The Authors. The Journal of Pathology published by John Wiley & Sons Ltd on behalf of Pathological Society of Great Britain and Ireland.

## Introduction

Cervical carcinoma is the third most common cause of cancer deaths in women worldwide. Most cases are squamous cell carcinomas (SCCs), which arise from precursor squamous intraepithelial lesions (SILs). Current treatments have not changed for decades and survival rates for advanced disease remain low 1. It is therefore important to unravel the molecular mechanisms of cervical SCC progression, in order to develop new therapies. A key feature of cervical squamous carcinogenesis is genomic instability, caused by deregulated expression of HPV oncogenes in proliferating epithelial cells 2,3. A genomic imbalance that is commonly selected in advanced cervical SCC is copy number gain of chromosome 5p 4,5.

One gene that is likely to drive selection of 5p gain is the oncostatin-M receptor (OSMR; located at 5p13.1), which is commonly up-regulated in cervical SCC and produces a significantly worse clinical outcome when over-expressed, independent of tumour stage 4. As a cell surface cytokine receptor, OSMR is a novel candidate for therapeutic targeting in cervical SCC. Recently, we showed that over-expressed OSMR enhanced the sensitivity of cervical SCC cells to the major ligand OSM, resulting in the induction of a pro-malignant phenotype. Direct cell effects included increased cell migration and invasiveness, without changes in cell proliferation 6. Using microarrays, we observed waves of specific gene induction over 72 h post-OSM stimulation in OSMR over-expressing cells. The most significant Gene Ontology category groups for the induced genes included angiogenesis, cell invasion/migration and signal transduction. Of particular interest were the 15 genes that also showed an association with OSMR levels in cervical SCC tissue samples, as identified from parallel global transcriptional profiling of 29 cases 6. These genes are listed in our previous publication (see Supplementary material, Table S9, in 6). They included tissue transglutaminase, also known as transglutaminase 2 (TGM2).

Transglutaminases are calcium-dependent crosslinking enzymes that catalyse post-translational protein modifications. TGM2 is the most diverse and ubiquitously expressed member of the family. Unlike other transglutaminases, TGM2 is a multifunctional protein, which has both enzymatic and non-enzymatic functions. These functions are closely related to its subcellular location and depend on the pathophysiological context 7,8. TGM2 is over-expressed in a range of cancer types, where it is associated with metastasis and decreased overall patient survival 9–13. Here we show, for the first time, that OSM–OSMR interactions strongly induce TGM2 expression in SCC cells. The over-expressed TGM2 interacts with integrin–*α*5*β*1 in the presence of fibronectin and induces a promalignant phenotype in cervical SCC cells. These *in vitro* findings are supported by evidence from tissue samples, that high TGM2 expression correlates with neoplastic progression in cervical and oral squamous epithelium, where TGM2 levels correlate with those of OSMR, integrin–*α*5*β*1 and fibronectin.

## Materials and methods

### Human tissue samples

All tissue samples were used with Local Research Ethics Committee approval. We analysed the following tissue samples and datasets:

- *Set 1*: 29 samples of cervical SCC, 32 samples of lesional epithelium microdissected from cervical high-grade squamous intraepithelial lesions (HSILs) and low-grade SILs (LSILs) and 12 samples of normal ectocervical squamous epithelium, which we previously used for microarray expression analysis 4,6. Raw data are available at Gene Expression Ommibus (GEO), Accession No. GSE27678. As part of this analysis, we studied the cervical SCCs that showed OSMR copy number gain (*n =* 13), comparing the cases that showed high-level OSMR over-expression (*n =* 3) with those that did not (*n =* 10).
- *Set 2*: 37 samples of cervical SCC, independent of those in set 1, in which *OSMR* gene copy number had previously been determined by tissue microarray (TMA) fluorescence *in situ* hybridization (FISH) 4. TMA sections were used for immunohistochemistry.
- *Sets 3–6*: four additional datasets of cervical and oral SCC samples with published gene expression profiles. The samples represented 32 cervical SCC plus 21 normal cervix (set 3) 5; 21 cervical SCC, seven HSIL plus 10 normal cervix (set 4) 14; 35 oral SCC plus six normal oral mucosa (set 5) 15; and 22 oral SCC plus five lymph node metastasis (set 6) 16 samples. All microarray data were obtained from the GEO database, with the Accession Nos GSE9750, GSE7803, GSE10121 and GSE2280, respectively.

### Immunohistochemistry

Sections (5 µm) were cut onto aminopropyltriethoxysilane-coated slides and processed for immunohistochemistry, as described 17. The primary antibody was mouse monoclonal anti-transglutaminase 2 (Lab Vision, Fremont, CA, USA; TG-100, IgG, 1:100). Briefly, primary antibody (100 µl) was applied in a humidified chamber at 4°C overnight in 1% bovine serum albumin (BSA) in Tris-buffered saline (TBS) containing 0.1% Triton X-100. The slides were then washed in TBS containing 0.025% Triton X-100 and incubated for 1 h with biotinylated goat anti-mouse secondary antibody (1:500; Dako, Glostrup, Denmark). A streptavidin–horseradish peroxidise (HRP) system (Dako) with the substrate diaminobenzidine was used to develop the stain and the slides were counterstained with Harris's haematoxylin. Negative controls were performed by omitting the primary antibody. We quantified the frequency of TGM2-positive cells, ie the percentage of tumour cells that stained positively for TGM2, regardless of intensity.

### Cell culture, OSM treatment and proliferation assays

We used the representative OSMR over-expressing cervical SCC cell lines, CaSki and SW756, as well as two non-OSMR over-expressing cervical SCC cell lines, MS751 and ME180 4,6. The cells were obtained from the American Type Culture Collection (ATCC-LGC, Middlesex, UK) and authenticated by short tandem repeat profiling 18. Cell culture and OSM treatment were performed as described 6. OSM was added at 10 ng/ml, in accordance with our previous studies examining long-term effects of OSM in tissue culture 6. For proliferation assays, cells were trypsinized and counted manually, using a Neubauer chamber. Dead cells were identified with the vital stain Trypan blue (Sigma, St. Louis, MO, USA).

### TGM2 enzymatic activity assay

The crosslinking activity of TGM2 was measured using the Specific Tissue Transglutaminase Colorimetric Microassay kit (TG2-CovTest, Covalab, Cambridge, UK), following the manufacturer's instructions. A standard curve of purified guinea pig TGM2 (specific activity 2200 U/mg) was used to calculate the enzyme activity in each sample.

### siRNA depletion

As part of multiple complementary approaches to minimize the possibility of non-specific observations, cells were transfected with short interfering RNA (siRNA), using Lipofectamine RNAiMAX (Invitrogen, Paisley, UK). Four TGM2-specific siRNAs (TGM2 ON-TARGETplus SMARTpool siRNA, Dharmacon, Lafayette, CO, USA) were used in a pool at 40 nm. Non-targeting siRNA and cyclophilin B siRNA (both Dharmacon) were used as negative and positive controls, respectively. The timings of OSM treatment and transfection were optimized to maximize silencing of OSM-induced TGM2. Based on this work (data not shown), OSM was added 6 h after siRNA transfection.

### TGM2 over-expression

We used a TGM2–pSG5 expression construct (Agilent Technologies, Santa Clara, CA, USA), kindly provided by Dr In-Gyu Kim, Seoul, Korea. Cells were transiently transfected with 2 µg construct by nucleofection (Amaxa Nucleofector, Lonza, Basel, Switzerland), using 1 × 10^6^ cells/transfection for SW756 and 2 × 10^6^ cells/transfection for CaSki. Control transfections were performed simultaneously, using 2 µg pmaxGFP expression vector (Amaxa Nucleofector). We compared cells expressing both TGM2 and GFP with those expressing GFP alone.

### Cell migration assay

Cells transfected with appropriate siRNAs or over-expression constructs were plated into fibronectin-coated two-chamber migration assay slides (Nunc, Roskilde, Denmark) at 3 × 10^5^ cells/chamber. After 24 h, the medium was temporarily removed and a wound created by a gentle scratch with a sterile 200 µl pipette tip. The cells were washed with phosphate-buffered saline (PBS) immediately and the medium replaced. Wound healing was monitored using time-lapse microscopy (Axiovert Tm200, Zeiss, Thornwood, NY, USA), with images captured every 5 min over a period of 24 h. Average migration velocity for individual cells in each experiment was calculated (Volocity 3D Image Analysis, Perkin-Elmer, Waltham, MA, USA) from 30 randomly chosen cells. This number of cells gave a statistical power > 0.95 for detecting a significant difference (*p <* 0.05) between experimental and control samples (see Supplementary material, Table S1; http://biostat.mc.vanderbilt.edu/PowerSampleSize). All experiments were performed in triplicate and the mean values used to indicate migration velocities.

### Cell invasion assay

Invasion assays were performed using the Cultrex Basement Membrane Extract (BME) Cell Invasion Assay (R&D Systems, Minneapolis, MN, USA), following the manufacturer's instructions; 0.5 × 10^6^ OSM-stimulated/unstimulated control-treated or siRNA-treated cells were seeded in the top chamber at the start of each assay. Standard curves for each SCC cell line were used to convert fluorescence values into cell number. All experiments were performed in triplicate and the mean values used to indicate invasion.

### Western blotting

Whole-cell lysates were subjected to SDS–PAGE and proteins transferred onto polyvinylidene fluoride membranes. The following primary antibodies were used: TGM2 (Lab Vision; 1:1600 and CST, Danvers, MA, USA; 1:1000); Fak (1:1000; CST); OSMR*β* (1:500; Santa Cruz Biotechnology, Dallas, TX, USA); integrin–*α*5 (1:2000), integrin–*β*1 (1:1000) and P-Fak (Y397, 1:1000) (all BD Biosciences, San Jose, CA, USA); and *β*-actin (1:100000; Abcam, Cambridge, UK). Secondary HRP-conjugated antibodies (1:1000) were from Dako. Luminograms were obtained with the Amersham Enhanced Chemiluminescence Detection Kit (GE Healthcare, Uppsala, Sweden) and densitometry performed using FluorChem-9900 imaging system software (Alpha-Innotech, San Leandro, CA, USA).

### Real-time quantitative PCR

Relative mRNA transcript levels were measured in triplicate using QuantiTect One-Step SYBR-Green qRT–PCR (Qiagen, Crawley, UK), following reverse transcription of 1 µg total RNA using QuantiTect Reverse Transcription (Qiagen). Primers were obtained from Qiagen (Quantitect primers) or PrimerBank (see Supplementary material, Table S2). Expression ratios were calculated using the comparative threshold cycle (*C*t) method 19 and normalized using three housekeeping genes; *ACTB*, *TBP* and *HMBS* 20.

### Flow cytometry

Live non-permeabilized cells detached with trypsin were stained at 4 °C with anti-TGM2 (1:100; Lab Vision) or anti-integrin–*α*5*β*1 (1:100, clone HA5; Millipore, Temecula, CA, USA) antibodies. After incubation with secondary Alexa 488 conjugated antibody (1:100; Molecular Probes, Leiden, The Netherlands), cells were analysed using a CyAn ADP flow cytometer (DakoCytomation, Glostrup, Denmark). Isotypic control IgG and an appropriate fluorochrome-conjugated secondary antibody were used in parallel as a negative control. The data were analysed using Summit 4.3 software (DakoCytomation).

### Immunofluorescence staining

Cells were seeded on fibronectin-coated coverslips, treated with OSM for 48 h and fixed using 4% paraformaldehyde for 10 min at room temperature. They were stained by immunofluorescence (IF) as described 17, using an anti-TGM2 rabbit monoclonal antibody (1:100; CST) and an anti-integrin–*α*5 mouse monoclonal antibody (1:100; Abcam). Alexa 488 conjugated goat anti-rabbit and Alexa 594 conjugated goat anti-mouse (1:1000; Molecular Probes) were used as secondary antibodies, with Hoechst 33258 (Sigma) nuclear counterstaining. Cells were examined by confocal laser-scanning microscopy (Zeiss LSM 510).

### Immunoprecipitation

Cells were washed with PBS and lysed in ice-cold RIPA buffer containing 1% Triton X-100, 0.5% sodium deoxycholate, 0.1% SDS, 150 mm NaCl, 50 mm Tris–Cl, pH 7.5, and protease and phosphatase inhibitor cocktail tablets (Roche, Penzberg, Germany). The lysates were incubated for 15 min at 4 °C on a rotating wheel and precleared by centrifugation at 13 000 rpm for 10 min. Non-specific binding was reduced by pre-incubating the lysates with protein G-Sepharose (GE Healthcare) for 20 min at 4 °C. Total cellular protein, 1 or 2 mg (from SW756 or CaSki, respectively) were immunoprecipitated overnight at 4 °C with 10 µl anti-integrin–*α*5*β*1 antibody (clone JBS5, Millipore) or normal mouse IgG (Millipore, used as negative control), followed by incubation with protein G-Sepharose for a further 4 h. The complexes were harvested by centrifugation, washed five times with PBS 0.1% Triton X-100, resolved in SDS–PAGE and immunoblotted.

### Pathway enrichment analysis

We performed comparative pathway analysis of two mRNA expression datasets obtained in our previous microarray profiling study 6. The first set was derived from cervical SCCs in sample set 1, that showed OSMR copy number gain and represented genes differentially expressed (*p <* 0.05) in the cases that showed high-level OSMR over-expression (*n =* 3), compared with those that did not (*n =* 10). This dataset was determined using Affymetrix U133 Plus 2.0 arrays (Santa Clara, CA, USA). The second set represented genes differentially expressed (*p <* 0.05) in CaSki or SW756 cells after OSM treatment, comparing cells at 12, 24 and 48 h time points with untreated (time 0 h) cells. This dataset was determined using Human Whole Genome 6 Expression Bead Chips v 2 (Illumina, San Diego, CA, USA). Pathway analysis was done using DAVID software 21,22, using platform-specific probe IDs. Background was set as *Homo sapiens* to avoid any platform bias. The categories searched were GO_BP, KEGG_PATHWAY, BIOCARTA and BBID. Functional annotation clusters and charts were saved and processed with PYTHON scripts, in order to match common information between the tissue sample and cell line annotation clusters. Pathways were considered to be shared between the clusters if the Benjamini adjusted *p* value was < 0.1 for both the tumour samples, and for either CaSki or SW756 at one or more of the time points analysed.

### Statistics

We used ANOVA, with a *post hoc* analysis by the Student–Newman–Keuls' test. Unless otherwise stated, results are expressed as mean ± standard error of the mean (SE).

## Results

### TGM2 is over-expressed and correlates with OSMR levels in human SCCs

**Figure 1:**
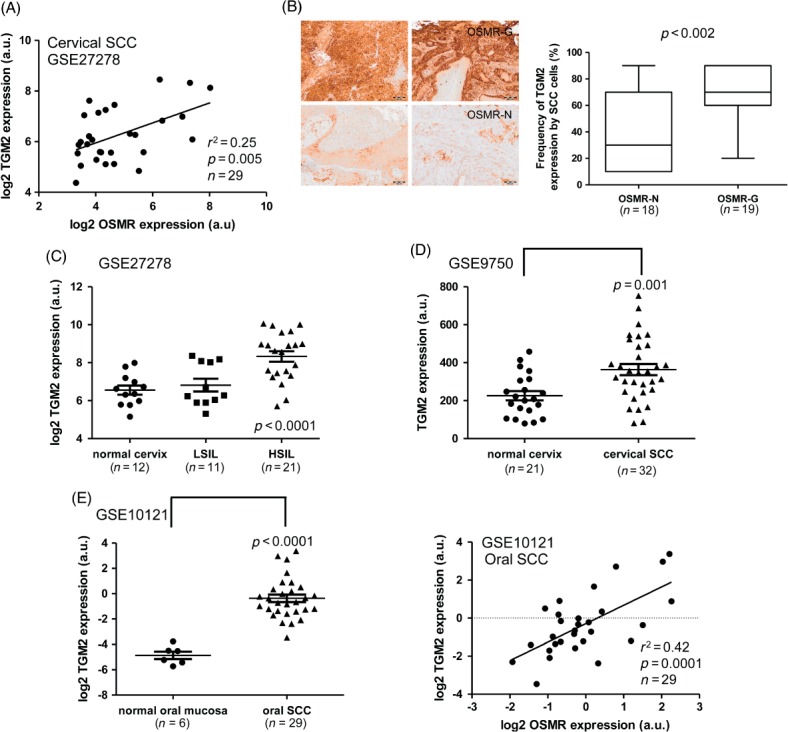
TGM2 expression in cervical and oral SCCs. (A) Linear regression analysis of mRNA levels of *TGM2* versus *OSMR* in microarray expression data for cervical SCC samples (set 1). (B) Correlation between TGM2 protein expression and OSMR copy number in cervical SCC samples (set 2). The left-hand panels show representative images from immunohistochemical staining for TGM2 in cervical SCC samples in which OSMR copy number gain was present (OSMR-G; top row) or absent (OSMR-N; bottom row); scale bars = 100 µm. The right-hand box plot shows the frequency of TGM2-positive SCC cells in samples where OSMR copy number gain was present (OSMR-G) or absent (OSMR-N): bar, median; box, IQR; whiskers, range. (C, D) Expression of *TGM2* mRNA in cervical neoplastic progression. *TGM2* mRNA expression in normal cervix samples, LSILs, HSILs and SCCs was determined from microarray data derived from sample sets 1 (C) and 3 (D). (E) Expression of TGM2 in oral SCC, based on expression microarray data for sample set 5. The left panel shows levels of *TGM2* mRNA in normal oral mucosa and oral SCC tissues, while the right panel shows linear regression analysis of *TGM2* mRNA levels versus *OSMR* mRNA levels in the oral SCC samples

In our previous study of sample set 1, TGM2 was significantly up-regulated in cervical SCC samples with OSMR over-expression, compared with those that lacked OSMR over-expression 6. There was no association between *OSMR* and *TGM2* gene copy number in this sample set, as assessed by comparative genomic hybridization (data not shown). Using linear regression analysis, we found that levels of *TGM2* mRNA correlated significantly with those of *OSMR* in all the set 1 SCC samples (*n =* 29; Figure 1A). The frequency of *TGM2*-positive cells, as determined by immunohistochemistry, was also significantly higher in sections of the 19 cervical SCC samples from set 2 that showed *OSMR* copy number gain, versus the 18 samples that lacked *OSMR* copy number gain (Figure 1B).

In view of these findings, we interrogated published data from gene expression profiling of cervical and oral SCC (sets 3–6), as well as our cervical SILs dataset (set 1). This showed that TGM2 expression correlated with disease progression in cervical tissue samples (Figure 1C, D). In set 1, TGM2 expression was higher in SILs than in normal cervix and was significantly increased in HSILs versus LSILs (Figure 1C). In set 3, TGM2 levels were increased in SCC samples compared to normal cervix (Figure 1D), and there was a positive correlation between levels of TGM2 and OSMR (see Supplementary material, Figure S1A). The correlation of TGM2 expression with disease progression was further validated in cervical sample set 4, in which expression levels increased progressively between normal cervix, HSILs and SCCs (see Supplementary material, Figure S1B).

Oral SCC samples (set 5) also showed over-expression of TGM2 compared to normal oral mucosa, with a positive correlation between levels of TGM2 and OSMR (Figure 1E). In an independent dataset of oral samples (set 6), TGM2 expression was higher in lymph node metastases than in the corresponding primary tumours and in a separate set of non-metastatic oral SCCs (see Supplementary material, Figure S1C). Again, there was a correlation between levels of TGM2 and OSMR in the oral SCCs and metastases from set 6 (see Supplementary material, Figure S1D).

### OSM-induced TGM2 enhances migration and invasion of cervical SCC cells

**Figure 2:**
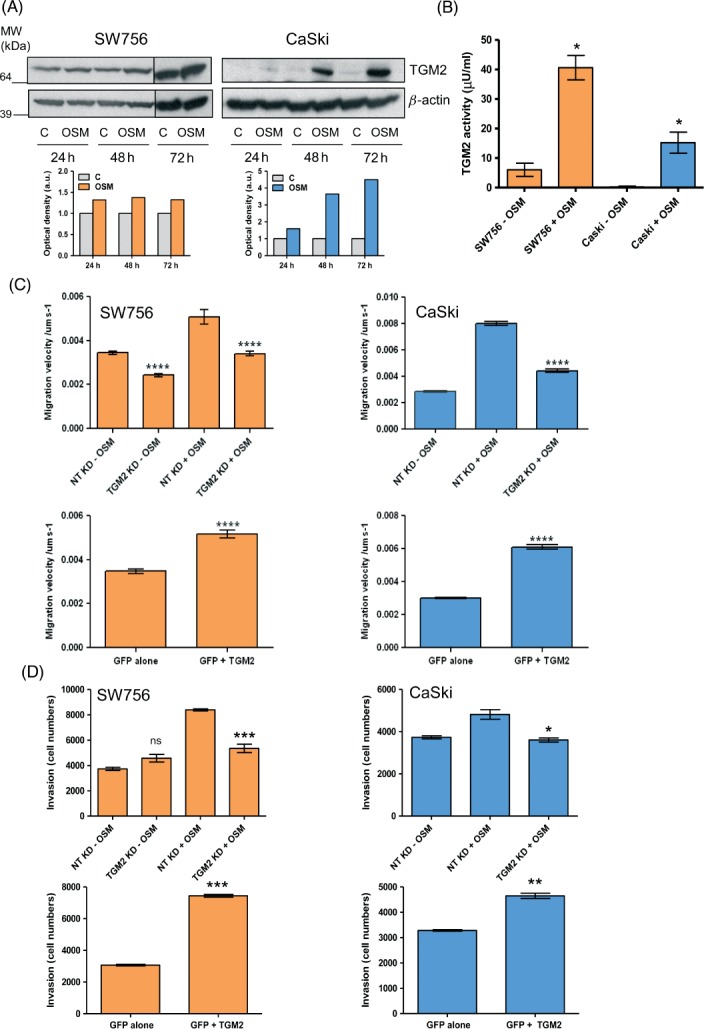
Effect of TGM2 depletion and over-expression on the migration and invasiveness of OSMR over-expressing cervical SCC cells. (A) Western blot showing levels of TGM2 protein in SW756 and CaSki at 24–72 h in control cells treated with vehicle only (C) or in OSM-treated cells (OSM). *β*-Actin (lower row) was used as the loading control. The graphs below each blot represent the densitometric analysis of protein levels. Results are expressed as optical density (arbitrary units), with control cells set as 1. (B) Levels of TGM2 enzymatic activity in SW756 and CaSki cells after 48 h in the absence (−OSM) or presence (+OSM) of OSM. (C, D) Quantification of cell migration velocity (C) and invasiveness through basement membrane extract (D). Each panel shows data for SW756 (left column) and CaSki (right column) following TGM2 depletion (top row) or TGM2 over-expression (bottom row). In the depletion experiments, cells were assayed after 48 h in the presence (+OSM) or absence (−OSM) of OSM. Cells with TGM2 knock-down (KD) were compared with control cells treated with non-targeting (NT) siRNA. As background levels of TGM2 were barely detectable in CaSki (see A), TGM2 depletion was only performed in OSM-treated CaSki cells. In the over-expression experiments, cells expressing TGM2 and GFP (GFP + TGM2) were compared with control cells expressing GFP alone: ns, non-significant; *p < 0.05; **p < 0.01; ***p < 0.001; ****p < 0.0001

As the evidence from human tissue samples suggested an important role for TGM2 in SCC progression, we examined the functional significance of TGM2 in cervical SCC cells *in vitro*. We used multiple complementary experimental approaches, based on gene depletion and over-expression, to minimize the possibility of non-specific observations. We focused on two representative OSMR-over-expressing cervical SCC cell lines, SW756 and CaSki 4,6. In both, treatment with OSM up-regulated TGM2 at the mRNA (see Supplementary material, Figure S2A) and protein levels (Figure 2A). After 48 h of OSM treatment, levels of TGM2 protein were induced by 360% in CaSki and by 140% in SW756 (in which basal levels of TGM2 were high). In addition, OSM significantly induced TGM2 enzymatic activity in both cell lines, as measured by a colorimetric assay (Figure 2B). This indicated that the OSM-induced TGM2 was functional and catalytically active. We observed mixed effects of exogenous OSM in the cervical SCC cell lines that did not over-express OSMR, MS751 and ME180 6. In MS751, TGM2 was induced after 24 h of OSM treatment, concomitant with up-regulation of OSMR (see Supplementary material, Figure S3). In contrast, in ME180 cells, TGM2 basal levels were undetectable and there was no induction by OSM (see Supplementary material, Figure S3).

In both SW756 and CaSki, a single treatment with TGM2-targeting pooled siRNAs depleted the TGM2 induced by OSM (see Supplementary material, Figure S4A). In our previous study 6, we found that OSM treatment of SW756 and CaSki increased cell migration and invasiveness but had no effect on proliferation. In both cell lines, depletion of TGM2 significantly reduced OSM-induced migration on fibronectin-coated surfaces, as well as invasion through extracellular matrix (Figure 2C, D, upper panels; see also Supplementary material, Figure S5). Conversely, ectopic TGM2 over-expression (see Supplementary material, Figure S4B–D) significantly increased the motility and invasiveness of both cell lines in the absence of OSM stimulation (Figure 2C, D, lower panels). TGM2 depletion and over-expression had no effect on cell proliferation and viability in the time frame of the invasion experiments (see Supplementary material, Figure S6). Together, these results demonstrated that TGM2 was a significant mediator of the pro-migratory and pro-invasive effects of OSM in OSMR over-expressing cervical SCC cells.

### TGM2 interacts with integrin–*α*5*β*1 in cervical SCC cells in an OSM-dependent manner

**Figure 3:**
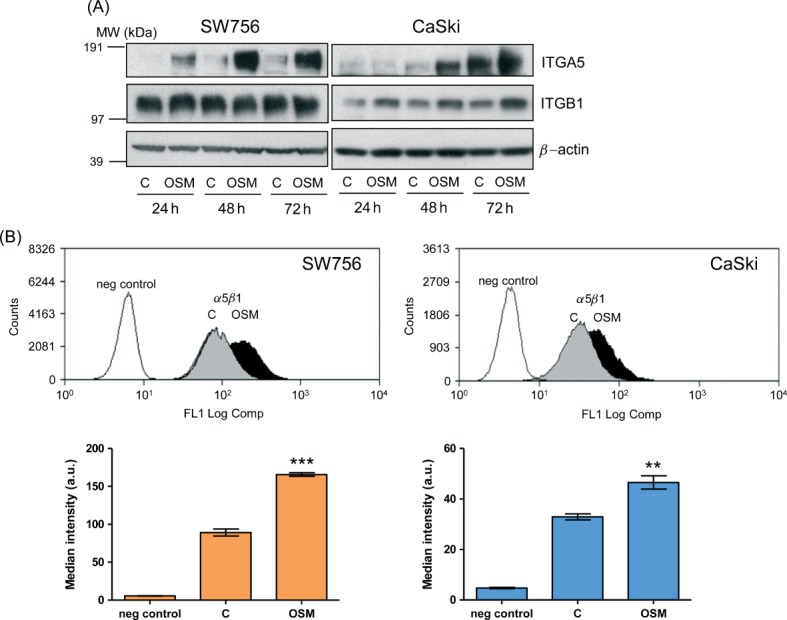
Integrin–*α*5*β*1 expression in cervical SCC cells. (A) Western blot analysis of total levels of integrin–*α*5 (ITGA5) and integrin–*β*1 (ITGB1) in SW756 and CaSki, comparing OSM-treated cells (OSM) with control cells treated with vehicle only (C); *β*-actin (lower row) was used as the loading control. (B) Flow-cytometric measurement of cell surface integrin–*α*5*β*1 in SW756 and CaSki cells, comparing cells treated with OSM for 48 h (OSM) with control cells treated with vehicle only (C). Negative control cells were stained with an isotype control IgG. In the graphs (bottom row) statistical comparisons were between OSM-treated and control-treated cells: **p < 0.01; ***p < 0.001

Another of the 15 genes that were induced by OSM in OSMR-over-expressing cervical SCC cells, and also associated with OSMR over-expression in cervical SCC tissues 6, was integrin–*α*5 (ITGA5). As TGM2 has been reported to interact with integrin–*α*5*β*1 in mesenchymal cells, leading to binding of the cognate ligand fibronectin 23,24, we examined interactions between OSMR, TGM2, integrin–*α*5*β*1 and fibronectin in cervical SCC cells. OSM increased integrin–*α*5 (*ITGA5*), integrin–*β*1 (*ITGB1*) and fibronectin (*FN1*) mRNA levels in both SW756 and CaSki (see Supplementary material, Figure S2). After 48 h of OSM treatment, integrin–*α*5 protein levels increased 7.1- and 2.9-fold for SW756 and CaSki, respectively (Figure 3A). Integrin–*β*1 protein expression was also induced by OSM in CaSki, with a 1.5-fold increase at 48 h. Basal levels of integrin–*β*1 protein in SW756 were very high and expression did not increase further upon OSM treatment (Figure 3A). In the OSMR non-over-expressing cervical SCC cells MS751 and ME180, OSM did not induce integrin–*α*5 or integrin–*β*1 (see Supplementary material, Figure S3).

**Figure 4:**
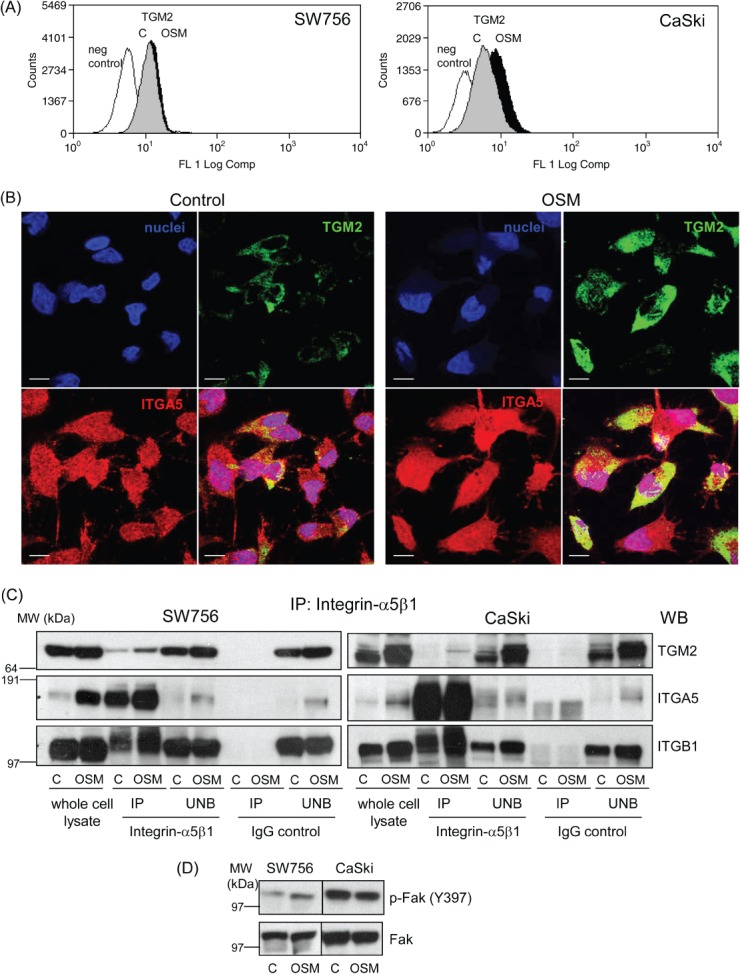
Interactions between TGM2 and integrin–*α*5*β*1 in cervical SCC cells. (A) Flow-cytometric quantification of cell-surface TGM2 expressed by SW756 and CaSki, comparing cells treated with OSM for 48 h (OSM) with control cells treated with vehicle only (C). Negative control cells were stained with an isotype control IgG. (B) Immunofluorescence for TGM2 (green) and integrin–*α*5 (red) in SW756 cells treated with vehicle (control) or OSM (48 h) for 48 h, showing focal co-localization (yellow); scale bars = 10 µm. (C) Immunoprecipitation showing physical interaction between TGM2 and integrin–*α*5*β*1 in SW756 and CaSki. Cells cultured on fibronectin-coated plates and treated with vehicle (C) or OSM (OSM) for 48 h were immunoprecipitated with anti-integrin–*α*5*β*1 antibodies or with IgG isotype-matched control antibodies. The immunoprecipitated (IP) and unbound (UNB) fractions, together with whole-cell lysates, were then analysed by western blotting, using antibodies against TGM2 (top row), integrin–*α*5 (ITGA5; middle row) and integrin–*β*1 (ITGB1; bottom row). The anti-integrin–*α*5*β*1 antibody precipitated TGM2 in both cell lines (cf lanes 3 and 4 versus 7 and 8 for each panel), with a stronger band for SW756 than for CaSki. Increased levels of precipitated protein were seen in OSM-treated cells (cf lanes 4 versus 3 in each panel). (D) Western blot showing levels of Y397-phosphorylated Fak (P-Fak) and total Fak in SW756 and CaSki cells cultured on fibronectin-coated plates and treated with OSM or vehicle (C) for 48 h

By flow cytometry, we observed that OSM treatment significantly increased expression of integrin–*α*5*β*1 dimers at the cell surface in both SW756 and CaSki (Figure 3B). The function of TGM2 relates to its subcellular location, with cell surface protein being responsible for interactions with integrins 8. Flow cytometry demonstrated the presence of TGM2 on the cell surface of both SW756 and CaSki (Figure 4A). OSM treatment increased the levels of membrane-associated TGM2, although the differences did not reach statistical significance. Confocal microscopy confirmed the cell membrane localization of TGM2 and showed focal co-localization of TGM2 and integrin–*α*5 in control and OSM-treated SW756 cells grown on fibronectin (Figure 4B).

Immunoprecipitation with an antibody against integrin–*α*5*β*1 confirmed that TGM2 interacted physically with integrin–*α*5*β*1 in SW756 and showed that OSM treatment strengthened the interaction (Figure 4C left, lanes 3 and 4). In CaSki, the interaction between TGM2 and integrin–*α*5*β*1 was only seen in OSM-stimulated cells (Figure 4C right, lanes 3 and 4), in agreement with the very low levels of TGM2 in unstimulated (control) cells (Figure 2A). For both cell lines, most of the TGM2 remained in the fraction unbound by the anti-integrin–*α*5*β*1 antibody (Figure 4C, lanes 5 and 6 in both panels). This was supported by our observation that most TGM2 is cytoplasmic in cervical SCC cells (Figure 4B) and was consistent with findings from mesenchymal cells 23. In the immunoprecipitation experiments, normal mouse IgG, used as an isotype control, failed to pull down TGM2 and integrin–*α*5*β*1 (Figure 4C, lanes 7 and 8 in both panels), confirming the specificity of the interaction between TGM2 and integrin–*α*5*β*1.

We next investigated whether OSM contributed to integrin-dependent signal transduction. In SW756 cells cultured on fibronectin, OSM treatment increased the levels of activated (Y397-phosphorylated) focal adhesion kinase (Fak), a known early marker of integrin activation that mediates cell adhesion, migration and invasion 25 (Figure 4D). Levels of activated Fak versus total Fak incresead 2.7-fold in OSM-treated cells, compared with control-treated cells. CaSki cells showed high basal levels of phosphorylated Fak (similar to SiHa cervical SCC cells grown on fibronectin 26), which were not increased further by OSM treatment (Figure 4D).

### Integrin–*α*5*β*1 and fibronectin are over-expressed and correlate with OSMR and TGM2 levels in human SCCs

**Figure 5:**
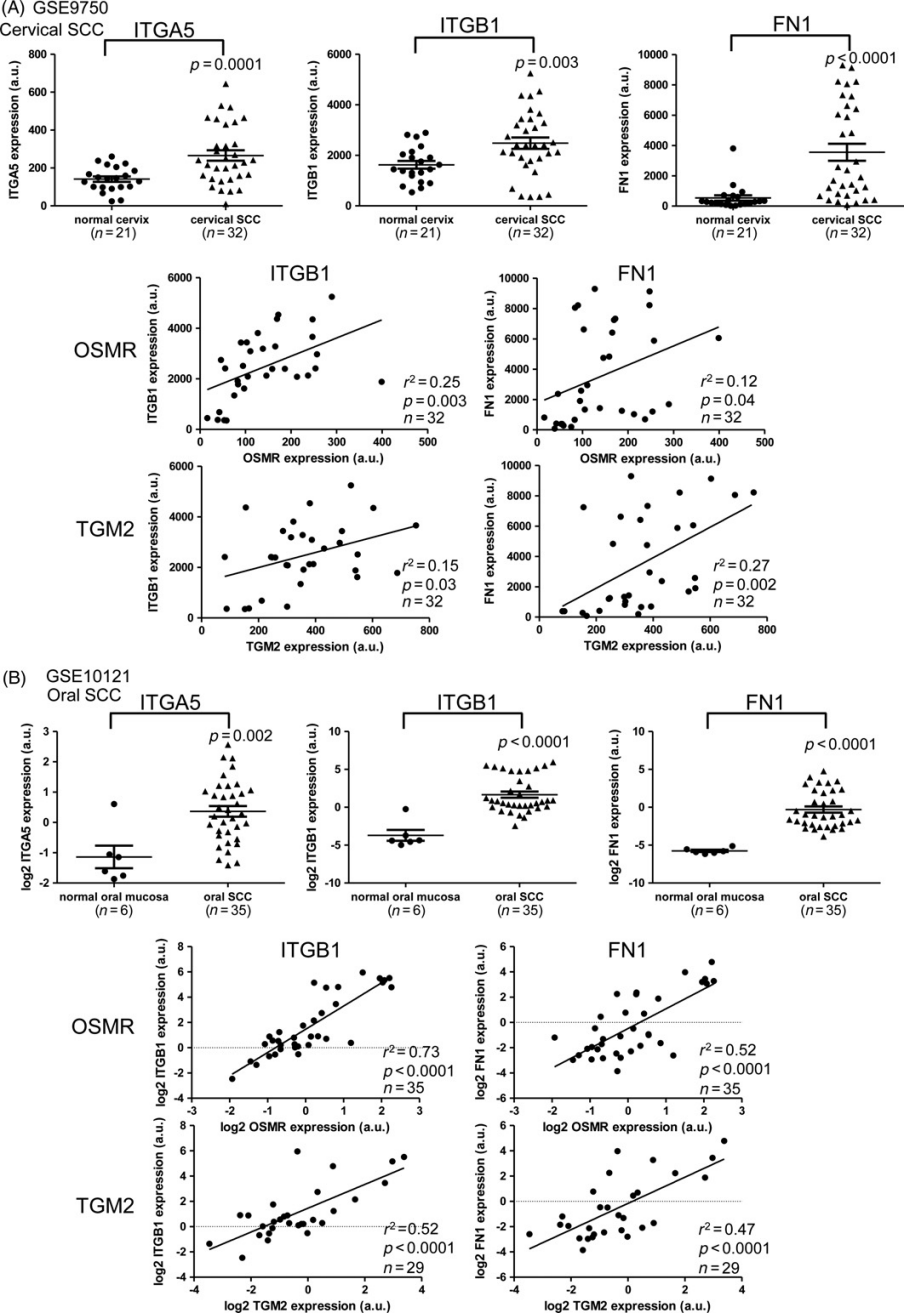
Integrin–*α*5*β*1 and fibronectin expression in cervical and oral SCCs. The panels show data derived from microarray analysis of (A) cervical SCCs (sample set 3) and (B) oral SCCs (sample set 5). In each panel, the top row shows levels of integrin–*α*5 (ITGA5; left), integrin–*β*1 (ITGB1; middle) and fibronectin (FN1; right) in normal mucosa and SCC tissues, while the middle and bottom rows show linear regression analysis of expression of ITGB1 and FN1 versus OSMR (middle) or TGM2 (bottom) in the SCC samples

By interrogating published datasets (sets 3 and 5), we found that integrin–*α*5*β*1 and fibronectin were significantly over-expressed in cervical and oral SCCs and that levels of integrin–*β*1 and fibronectin correlated with those of OSMR and TGM2 (Figure 5). These correlations were validated in our independent dataset of cervical SCCs (set 1), where integrin–*α*5 levels were also associated with OSMR expression (see Supplementary material, Figure S7, Table S3). In an independent dataset of oral SCCs (set 6), levels of integrin–*α*5*β*1 and fibronectin again correlated with those of OSMR, while integrin–*α*5 and fibronectin levels correlated with TGM2 expression (see Supplementary material, Figure S7).

Finally, we analysed pathways that were enriched in the OSMR-over-expressing versus non-over-expressing cervical SCCs from sample set 1, adopting the very stringent approach used in our previous study 6 (see Materials and methods). In total, 90 pathways were significantly enriched. Eight of the 21 top-ranking pathways (ranked by adjusted *p* value) were related to cell migration, adhesion and extracellular matrix (ECM) interactions (Table1). Four of these pathways were also enriched in OSM-treated CaSki or SW756 cells, compared with untreated cells. Together, these findings further supported our evidence of associations between OSMR, TGM2, integrin–*α*5*β*1 and fibronectin that promoted pro-malignant phenotypic changes in cervical SCC cells.

**Table 1:** Pathways enriched in OSMR over-expressing cervical SCCs

Ranking	Category term	GO or KEGG ID	Gene count	(%)	Fold enrichment	Adj *p*
1/90	Focal adhesion	hsa04510	40	3.97	2.71	1.55E-06
2/90	Cell motion*	GO:0006928	65	6.45	2.22	3.42E-06
11/90	Biological adhesion	GO:0022610	76	7.54	1.76	3.93E-04
14/90	Response to wounding*	GO:0009611	62	6.15	1.90	4.43E-04
15/90	Cell adhesion	GO:0007155	76	7.54	1.76	4.82E-04
16/90	Cell migration*	GO:0016477	39	3.87	2.29	5.77E-04
19/90	ECM–receptor interaction	hsa04512	19	1.88	3.08	2.18E-03
21/90	Cell motility*	GO:0048870	39	3.87	2.06	5.42E-03

In total, 90 categories were significantly enriched in the OSMR over-expressing cervical SCCs from sample set 1, compared with the non-over-expressing SCCs. The table shows the eight categories from the top 21 (ranked by adjusted *p* value) that supported our evidence of functionally significant interactions between OSMR and TGM2, integrin-*α*5*β*1 and fibronectin in cervical SCC cells.

* Pathways that were also up-regulated following OSM treatment of CaSki or SW756 cells.

Gene count, number of genes differentially expressed per category; (%), proportion of genes differentially expressed per category; Fold enrichment, ratio of the proportion of genes from each category in the differentially expressed genes versus the proportion in all genes on the array.

## Discussion

These combined tissue sample and *in vitro* data represent the first demonstration that TGM2 is an important mediator of the ligand-dependent pro-malignant effects of OSMR over-expression in cervical SCC cells. OSM is a member of the IL-6 cytokine family, and is known to signal through JAK–STAT pathways to activate context-dependent target genes 27. We previously demonstrated that OSM induced STAT3 activation in an OSMR-dependent manner in cervical SCC cells 4, and others have shown that STAT1 and STAT5 can also be activated by OSM–OSMR binding 27. Interestingly, there are putative STAT binding sites in the TGM2 promoter 28. Our data suggest that OSM–OSMR interactions may also be responsible for inducing TGM2 in other cell types, such as breast cancer cells, in which OSM promotes cell motility and metastatic behaviour and TGM2 has been associated with migration, invasion and metastasis 29–33. In support of this, TGM2 was identified by microarray analysis as one of the genes up-regulated in T47D breast cancer cells after OSM treatment 34.

Our analyses of multiple independent clinical sample sets demonstrate that expression of TGM2 increases during squamous neoplastic progression in the cervix and oral cavity. In the cervix, levels increased between normal cervix, SIL and SCC, while elevated levels were also seen in oral SCC, with higher expression in metastases compared to non-metastatic tumours. These findings are supported by previous immunohistochemical studies of clinical samples. TGM2 expression was higher in cervical SILs and SCCs than in normal cervical squamous epithelium 35, while high TGM2 expression in laryngeal SCC was associated with decreased overall survival in patients treated by surgery and radiotherapy 36.

Together, our results suggest the existence of a biologically significant OSMR/TGM2/integrin-α5β1 pathway in cervical and oral SCC. TGM2–integrin–fibronectin associations have previously been implicated in the cell motility and invasion of breast cancer cells 29. While integrin–*α*5*β*1 is usually expressed at low or undetectable levels in most adult epithelia, it is highly up-regulated in some tumours, including melanomas and lung carcinomas, where high expression levels correlate with advanced disease and reduced patient survival 37. Integrins control ECM remodelling, which is necessary for cancer cell migration and invasion. Indeed, over-expression of fibronectin and other ECM proteins has been associated with cancer progression and metastasis 38. Individually, integrin–*α*5, fibronectin and other ECM proteins have been shown to be over-expressed in cervical SCC samples compared with normal cervical epithelium 39–41. Fibronectin and other ECM proteins were also up-regulated in oral SCC, where they were associated with lymph node metastasis 42. These data reinforce our evidence that the TGM2–integrin–*α*5*β*1–fibronectin interactions that are activated by OSMR stimulation in SCC cells are of biological and clinical significance.

Our findings further strengthen the interest in antibody-based inhibition of OSM–OSMR binding as a novel cancer therapeutic strategy. Humanized antibodies against OSM are showing considerable promise in treating rheumatoid arthritis, where they are able to inhibit lymphocyte migration and reduce inflammation 43. Our accumulated data ( 4,6 and the present study) suggest that analogous benefits will also be obtained in cervical SCC (and potentially other tumour types where OSMR is over-expressed), by reducing tumour cell migration and invasion. It may be possible to increase therapeutic efficacy through simultaneous targeting of other points in the OSMR/TGM2/integrin-α5β1/fibronectin pathway. For example, TGM2 inhibitors have been shown to be effective in preclinical models of glioblastoma, where they cooperated with chemotherapy in reducing tumour growth by disrupting fibronectin assembly 44, while small molecules and antibodies targeting integrin–*α*5*β*1 are currently being used in clinical trials to block tumour angiogenesis 37. *In vivo* studies using appropriate preclinical models are now required to test the clinical benefits of targeting this functionally significant pathway in cervical SCC cells.

## SUPPLEMENTARY MATERIAL ON THE INTERNET

Figure S1. Validation of TGM2 correlations with OSMR expression and squamous neoplastic progression in the cervix and oral mucosa

Figure S2. Expression of TGM2, integrin–*α*5*β*1 and fibronectin in OSMR over-expressing cervical SCC cells 24–72 h after OSM treatment

Figure S3. Expression of TGM2, integrin–*α*5*β*1 and OSMR in non-OSMR-over-expressing cervical SCC cells 24–72 h after OSM treatment

Figure S4. Levels and activity of TGM2 in SW756 and CaSki after TGM2 depletion and over-expression

Figure S5. Wound healing in SW756 and CaSki cells

Figure S6. Effect of TGM2 depletion and over-expression on the proliferation and cell viability of OSMR-over-expressing cervical SCC cells

Figure S7. Validation of correlations between levels of integrin–*α*5*β*1 and fibronectin versus OSMR and TGM2

Table S1. Power calculations for cell migration experiments

Table S2. Primers used for real-time quantitative PCR

Table S3. Differential expression of selected genes in cervical SCCs according to OSMR levels

## Supporting Information

Figure S1. Validation of TGM2 correlations with OSMR expression and squamous neoplastic progression in the cervix and oral mucosa. (A) Linear regression analysis of mRNA levels of *TGM2* versus *OSMR* in the cervical SCC samples from set 3. (B) Expression levels of *TGM2* mRNA in normal cervix and in cervical HSIL and SCCs from sample set 4. (C) Levels of *TGM2* mRNA in sample set 6, comparing primary oral SCCs with their corresponding lymph node (LN) metastases (left panel) and the LN metastases with non-metastatic oral SCCs (right panel). (D) Linear regression analysis of *TGM2* versus *OSMR* mRNA levels in the oral SCC and LN metastasis samples from set 6

Figure S2. Expression of TGM2, integrin–*α*5*β*1 and fibronectin in OSMR-over-expressing cervical SCC cells 24–72 h after OSM treatment. Messenger RNA levels of TGM2 (A), integrin–*α*5 (ITGA5) (B), integrin–*β*1 (ITGB1) (C) and fibronectin (FN1) (D), as determined by quantitative PCR in SW756 (left) and CaSki (right) after 24–72 h of treatment with OSM. Data are referenced to control cells treated with vehicle only (C) at each time point. The graphs show results from one representative experiment of three performed

Figure S3. Expression of TGM2, integrin–*α*5*β*1 and OSMR in non-OSMR-over-expressing cervical SCC cells 24–72 h after OSM treatment. Western blot showing levels of TGM2, integrin–*α*5 (ITGA5), integrin–*β*1 (ITGB1) and OSMR protein in MS751 and ME180 at 24–72 h, comparing OSM-treated cells (OSM) with control cells treated with vehicle only (C). *β*-Actin (lower row) was used as the loading control

Figure S4. Levels and activity of TGM2 in SW756 and CaSki after TGM2 depletion and over-expression. (A) Western blots showing levels of TGM2 protein in OSM-treated and untreated SW756 and CaSki cells, 48 h after transfection with pooled siRNA duplexes targeting TGM2. *β*-Actin was used as the loading control. The graphs below each blot show the densitometric analysis of protein levels. Results are expressed as optical density (arbitrary units), with control cells set as 1. (B–D) Levels of TGM2 protein (B), mRNA (C) and enzyme activity (D) in SW756 and CaSki cells after transfection with the TGM2 over-expression vector. Data in (B, D) were obtained 48 h after transfection, while those in (C) were obtained at 24 and 48 h. The graphs in (B) show densitometric analysis of the protein levels, as described for (A): **p <* 0.05; ***p <* 0.01; ****p <* 0.001; *****p <* 0.0001 versus wt cells

Figure S5. Wound-healing in SW756 and CaSki cells. The panels show representative images of wound healing over a 6 h period by SW756 and CaSki cells treated with non-targeting (NT) or TGM2-targeting siRNA duplexes (TGM2 KD). (A) SW756 in the absence of OSM; (B, C) SW756 and CaSki, respectively, in the presence of OSM. As background levels of TGM2 were barely detectable in CaSki (see FigureA), TGM2 depletion was only performed in OSM-treated CaSki cells: scale bars = 100 µm

Figure S6. Effect of TGM2 depletion and over-expression on the proliferation and viability of OSMR-over-expressing cervical SCC cells. Quantification of cell proliferation (A) and cell death (B) at 24 and 48 h after transfection with pooled siRNA duplexes targeting TGM2, or with the TGM2 over-expression vector. Each panel shows data for SW756 (left column) and CaSki (right column), following TGM2 depletion (top row) or TGM2 over-expression (bottom row). In the depletion experiments, cells with TGM2 knockdown (KD) were compared with control cells treated with non-targeting (NT) siRNA, and in the over-expression experiments, cells expressing TGM2 and GFP (GFP + TGM2) were compared with control cells expressing GFP alone. Graphs show results from one representative experiment of two performed for TGM2 depletion and three performed for TGM2 over-expression

Figure S7. Validation of correlations between levels of integrin–*α*5*β*1 and fibronectin versus OSMR and TGM2. (A) Linear regression analysis of mRNA levels of *TGM2* versus integrin–*β*1 (*ITGB1*) and fibronectin (*FN1*) in cervical SCCs (set 1); for OSMR correlations in this sample set, see Table S2. (B) Linear regression analysis of mRNA levels of *OSMR* (top row) and *TGM2* (bottom row) versus levels of integrin–*α*5 (*ITGA5*), integrin–*β*1 (*ITGB1*) and fibronectin (*FN1*) in oral SCCs and LN metastases (set 6)

Table S1. Power calculations for cell migration experiments. *n*, number of test cells; *α*, the type I error probability; *m*, ratio of control to experimental cells; *δ*, difference in population means; *σ*, within-group standard deviation; NT, non-targeting; KD, knockdown. As background levels of TGM2 were barely detectable in CaSki (see FigureA), TGM2 depletion was only performed in OSM-treated CaSki cells.

Table S2. Primers used for real-time quantitative PCR. NA, not applicable.

Table S3. Differential expression of OSMR–TGM2–integrin–*α*5*β*1–fibronectin pathway genes in cervical SCCs, according to OSMR levels. For each gene, the log2 ratio refers to expression levels in cervical SCCs with OSMR over-expression, compared with those without over-expression. The data were derived from our previous microarray profiling of sample set 1. Probe sets are ranked by *p* value.
