# Supplementary material for: Tissue transglutaminase mediates the pro-malignant effects of oncostatin M receptor over-expression in cervical squamous cell carcinoma
Source: J Pathol. 2013 Sep 10;231(2):168–79. doi: 10.1002/path.4222 (PMC4288975; doi:10.1002/path.4222)
Supplement: Supplementary file 1 — Figure S1. Validation of TGM2 correlations with OSMR expression and squamous neoplastic progression in the cervix and oral mucosa. (A) Linear regression analysis of mRNA levels of TGM2 versus OSMR in the cervical SCC samples from set 3. (B) Expression levels of TGM2 mRNA in normal cervix and in cervical HSIL and SCCs from sample set 4. (C) Levels of TGM2 mRNA in sample set 6, comparing primary oral SCCs with their corresponding lymph node (LN) metastases (left panel) and the LN metastases with non-metastatic oral SCCs (right panel). (D) Linear regression analysis of TGM2 versus OSMR mRNA levels in the oral SCC and LN metastasis samples from set 6 [file path0231-0168-sd1.pptx]

## Slide 1
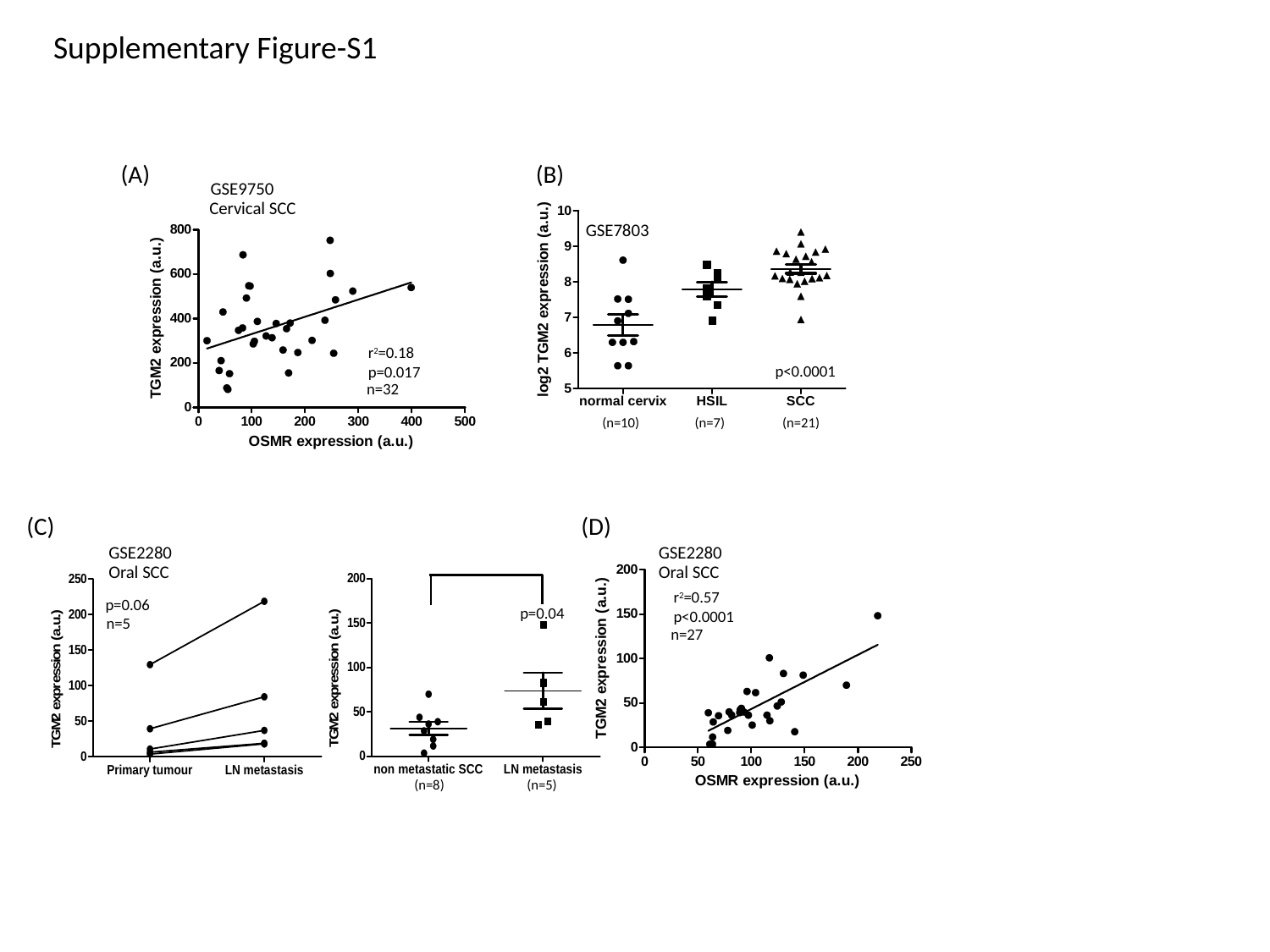

Supplementary Figure-S1
(A)
(B)
GSE9750
GSE7803
p<0.0001
(n=10)
(n=7)
(n=21)
Cervical SCC
r2=0.18
p=0.017
n=32
(C)
(D)
GSE2280
GSE2280
Oral SCC
Oral SCC
p=0.04
r2=0.57
p<0.0001
p=0.06
n=5
n=27
(n=8)
(n=5)
