# Supplementary figures and images for: Tissue transglutaminase mediates the pro-malignant effects of oncostatin M receptor over-expression in cervical squamous cell carcinoma
Source: J Pathol. 2013 Sep 10;231(2):168–79. doi: 10.1002/path.4222 (PMC4288975; doi:10.1002/path.4222)

## Slide 1
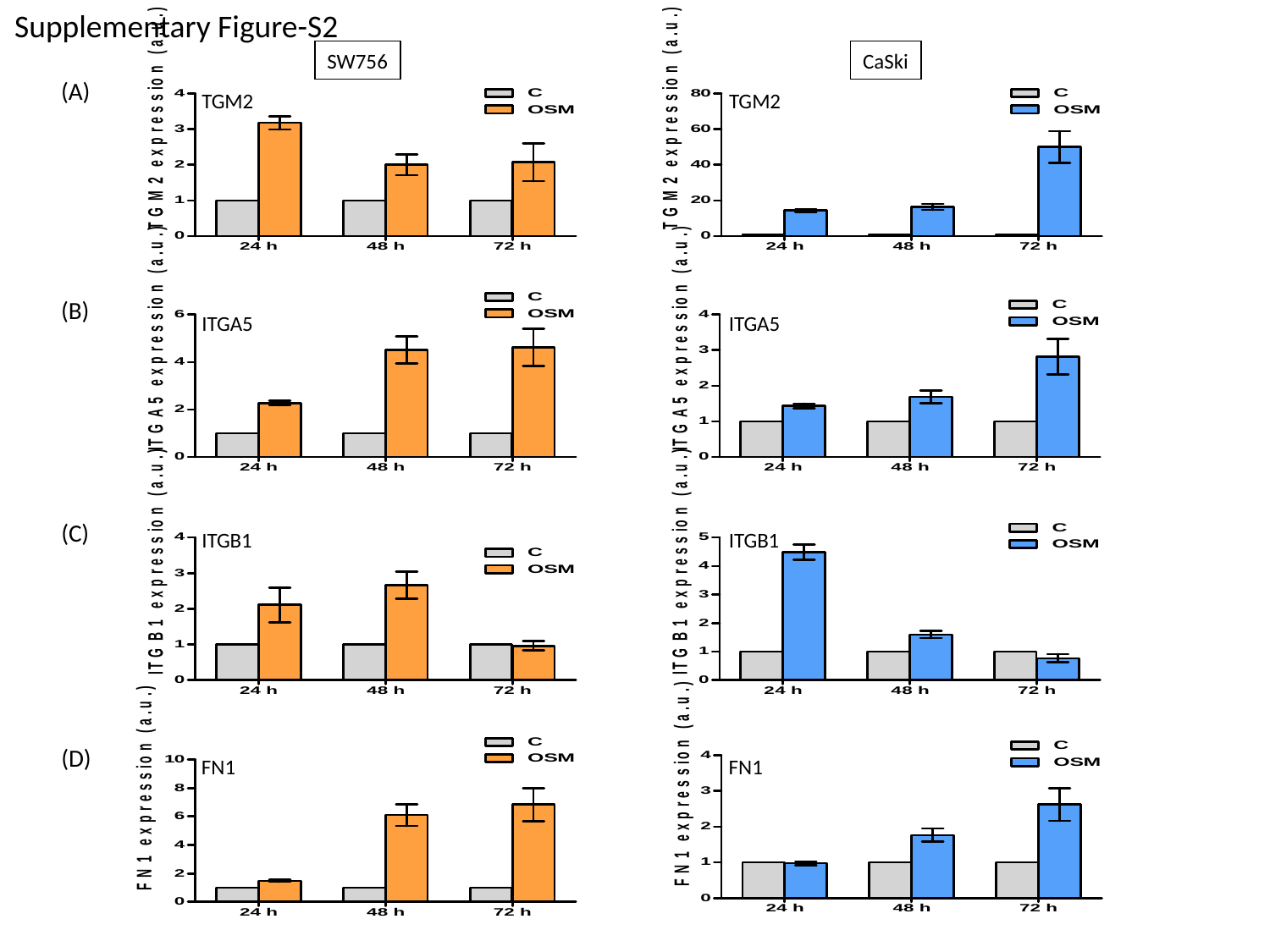

Supplementary Figure-S2
SW756
CaSki
(A)
TGM2
TGM2
(B)
ITGA5
ITGA5
(C)
ITGB1
ITGB1
(D)
FN1
FN1

Supplement: Supplementary file 2 — Figure S2. Expression of TGM2, integrin–α5β1 and fibronectin in OSMR-over-expressing cervical SCC cells 24–72 h after OSM treatment. Messenger RNA levels of TGM2 (A), integrin–α5 (ITGA5) (B), integrin–β1 (ITGB1) (C) and fibronectin (FN1) (D), as determined by quantitative PCR in SW756 (left) and CaSki (right) after 24–72 h of treatment with OSM. Data are referenced to control cells treated with vehicle only (C) at each time point. The graphs show results from one representative experiment of three performed [file path0231-0168-sd2.pptx]

## Slide 1
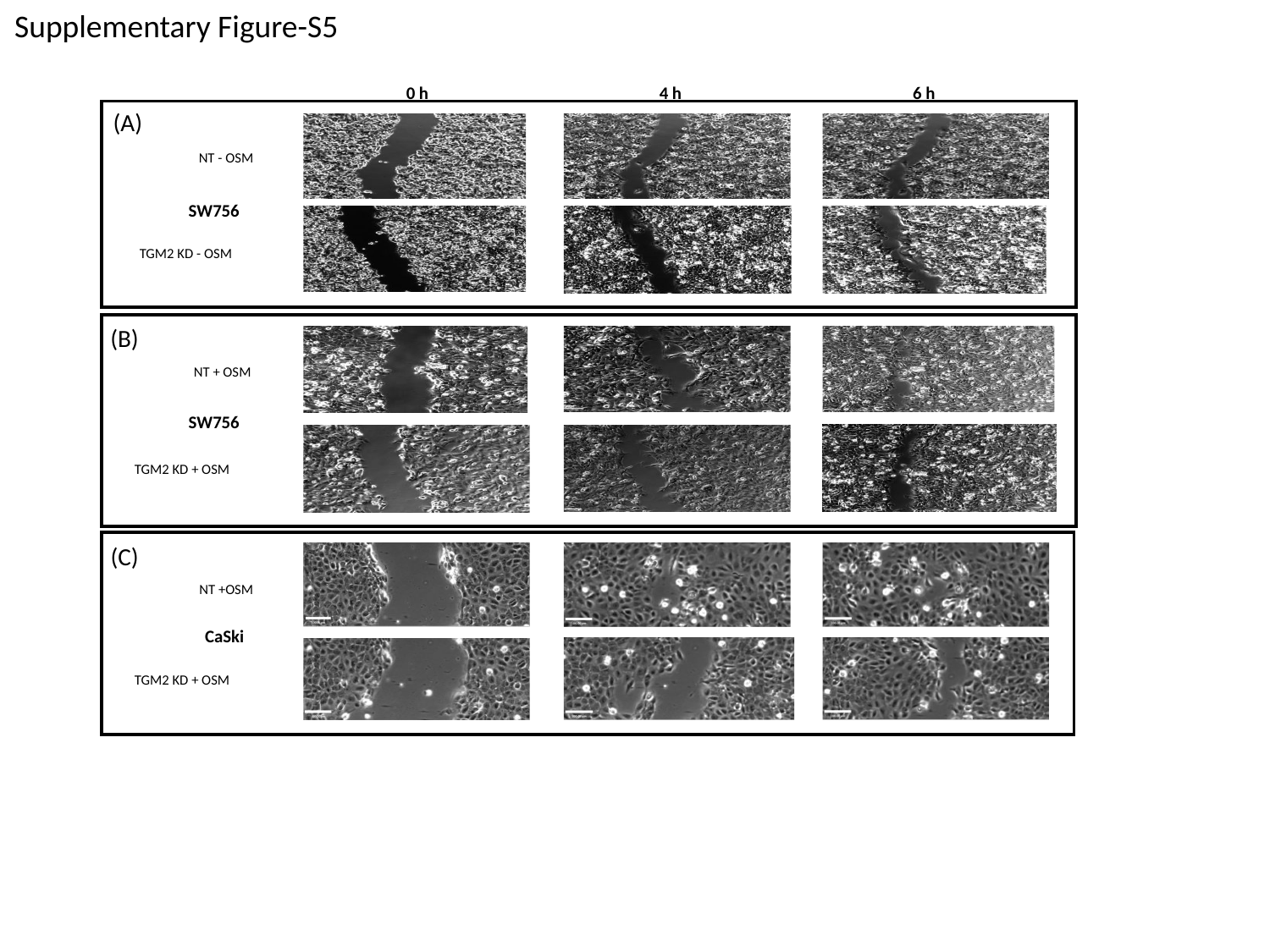

Supplementary Figure-S5
0 h
4 h
6 h
(A)
NT - OSM
SW756
TGM2 KD - OSM
(B)
NT + OSM
SW756
TGM2 KD + OSM
(C)
NT +OSM
CaSki
TGM2 KD + OSM

Supplement: Supplementary file 5 — Figure S5. Wound-healing in SW756 and CaSki cells. The panels show representative images of wound healing over a 6 h period by SW756 and CaSki cells treated with non-targeting (NT) or TGM2-targeting siRNA duplexes (TGM2 KD). (A) SW756 in the absence of OSM; (B, C) SW756 and CaSki, respectively, in the presence of OSM. As background levels of TGM2 were barely detectable in CaSki (see FigureA), TGM2 depletion was only performed in OSM-treated CaSki cells: scale bars = 100 µm [file path0231-0168-sd5.pptx]

## Slide 1
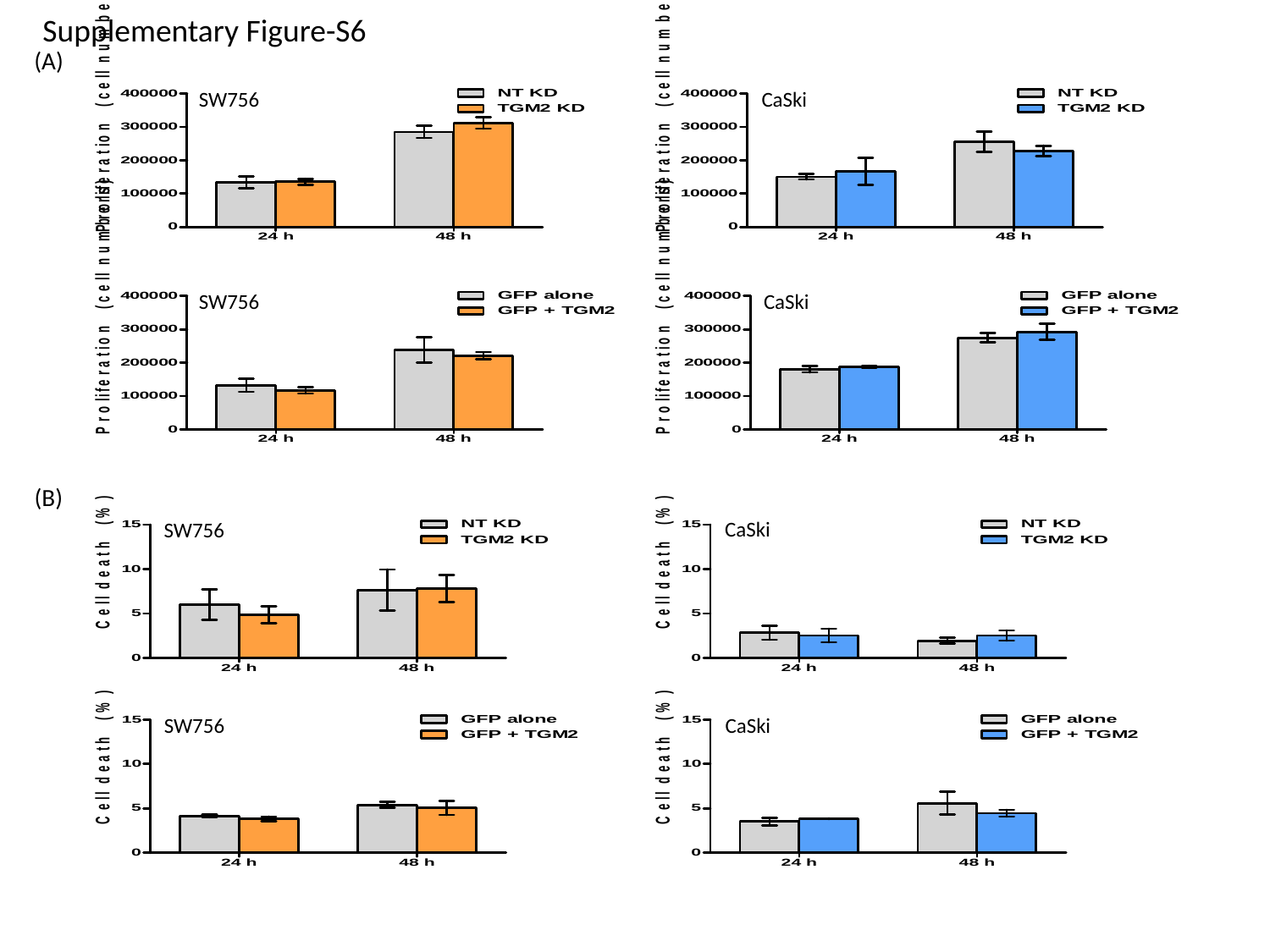

Supplementary Figure-S6
(A)
SW756
CaSki
SW756
CaSki
(B)
CaSki
SW756
SW756
CaSki

Supplement: Supplementary file 6 — Figure S6. Effect of TGM2 depletion and over-expression on the proliferation and viability of OSMR-over-expressing cervical SCC cells. Quantification of cell proliferation (A) and cell death (B) at 24 and 48 h after transfection with pooled siRNA duplexes targeting TGM2, or with the TGM2 over-expression vector. Each panel shows data for SW756 (left column) and CaSki (right column), following TGM2 depletion (top row) or TGM2 over-expression (bottom row). In the depletion experiments, cells with TGM2 knockdown (KD) were compared with control cells treated with non-targeting (NT) siRNA, and in the over-expression experiments, cells expressing TGM2 and GFP (GFP + TGM2) were compared with control cells expressing GFP alone. Graphs show results from one representative experiment of two performed for TGM2 depletion and three performed for TGM2 over-expression [file path0231-0168-sd6.pptx]

## Slide 1
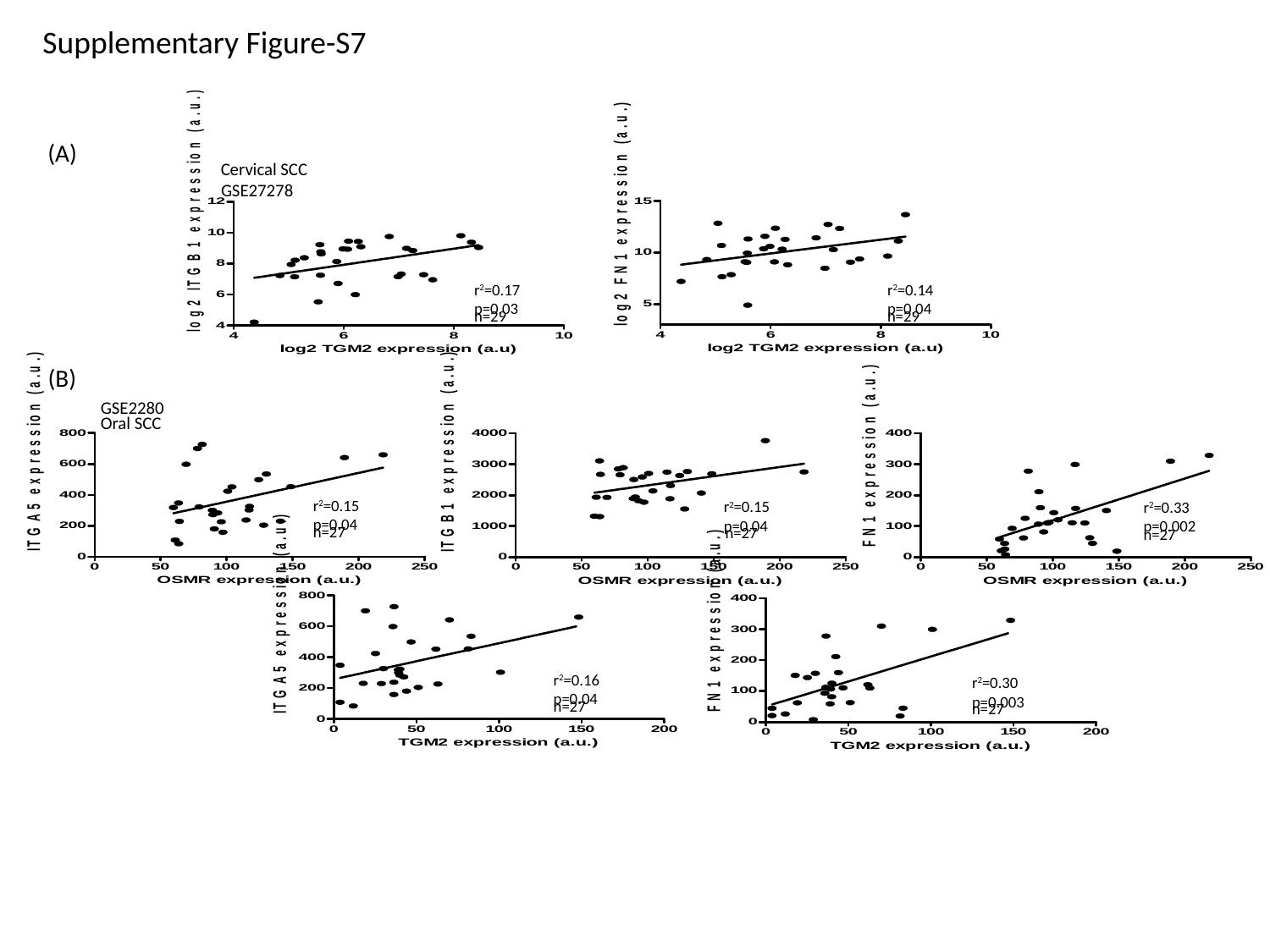

Supplementary Figure-S7
(A)
Cervical SCC
GSE27278
r2=0.17
p=0.03
r2=0.14
p=0.04
n=29
n=29
(B)
GSE2280
Oral SCC
r2=0.15
p=0.04
r2=0.15
p=0.04
r2=0.33
p=0.002
n=27
n=27
n=27
r2=0.16
p=0.04
n=27
r2=0.30
p=0.003
n=27

Supplement: Supplementary file 7 — Figure S7. Validation of correlations between levels of integrin–α5β1 and fibronectin versus OSMR and TGM2. (A) Linear regression analysis of mRNA levels of TGM2 versus integrin–β1 (ITGB1) and fibronectin (FN1) in cervical SCCs (set 1); for OSMR correlations in this sample set, see Table S2. (B) Linear regression analysis of mRNA levels of OSMR (top row) and TGM2 (bottom row) versus levels of integrin–α5 (ITGA5), integrin–β1 (ITGB1) and fibronectin (FN1) in oral SCCs and LN metastases (set 6) [file path0231-0168-sd7.pptx]
