# Supplementary material for: Tissue transglutaminase mediates the pro-malignant effects of oncostatin M receptor over-expression in cervical squamous cell carcinoma
Source: J Pathol. 2013 Sep 10;231(2):168–79. doi: 10.1002/path.4222 (PMC4288975; doi:10.1002/path.4222)
Supplement: Supplementary file 3 — Figure S3. Expression of TGM2, integrin–α5β1 and OSMR in non-OSMR-over-expressing cervical SCC cells 24–72 h after OSM treatment. Western blot showing levels of TGM2, integrin–α5 (ITGA5), integrin–β1 (ITGB1) and OSMR protein in MS751 and ME180 at 24–72 h, comparing OSM-treated cells (OSM) with control cells treated with vehicle only (C). β-Actin (lower row) was used as the loading control [file path0231-0168-sd3.pptx]

## Slide 1
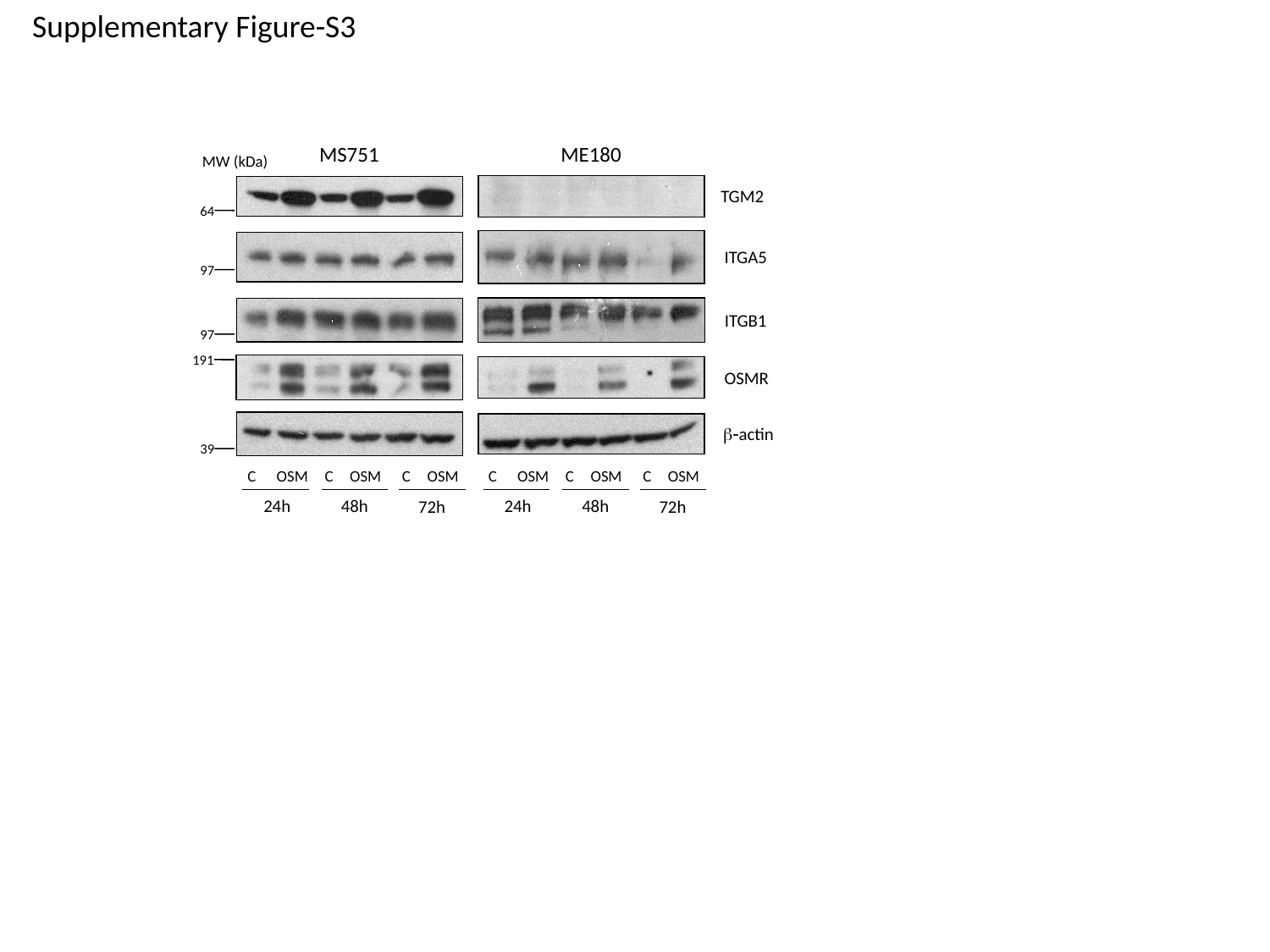

Supplementary Figure-S3
MS751
ME180
MW (kDa)
TGM2
64
ITGA5
97
ITGB1
97
191
OSMR
b-actin
39
C
OSM
C
OSM
C
OSM
24h
48h
72h
C
OSM
C
OSM
C
OSM
24h
48h
72h
