# Supplementary material for: Tissue transglutaminase mediates the pro-malignant effects of oncostatin M receptor over-expression in cervical squamous cell carcinoma
Source: J Pathol. 2013 Sep 10;231(2):168–79. doi: 10.1002/path.4222 (PMC4288975; doi:10.1002/path.4222)
Supplement: Supplementary file 4 — Figure S4. Levels and activity of TGM2 in SW756 and CaSki after TGM2 depletion and over-expression. (A) Western blots showing levels of TGM2 protein in OSM-treated and untreated SW756 and CaSki cells, 48 h after transfection with pooled siRNA duplexes targeting TGM2. β-Actin was used as the loading control. The graphs below each blot show the densitometric analysis of protein levels. Results are expressed as optical density (arbitrary units), with control cells set as 1. (B–D) Levels of TGM2 protein (B), mRNA (C) and enzyme activity (D) in SW756 and CaSki cells after transfection with the TGM2 over-expression vector. Data in (B, D) were obtained 48 h after transfection, while those in (C) were obtained at 24 and 48 h. The graphs in (B) show densitometric analysis of the protein levels, as described for (A): *p < 0.05; **p < 0.01; ***p < 0.001; ****p < 0.0001 versus wt cells [file path0231-0168-sd4.pptx]

## Slide 1
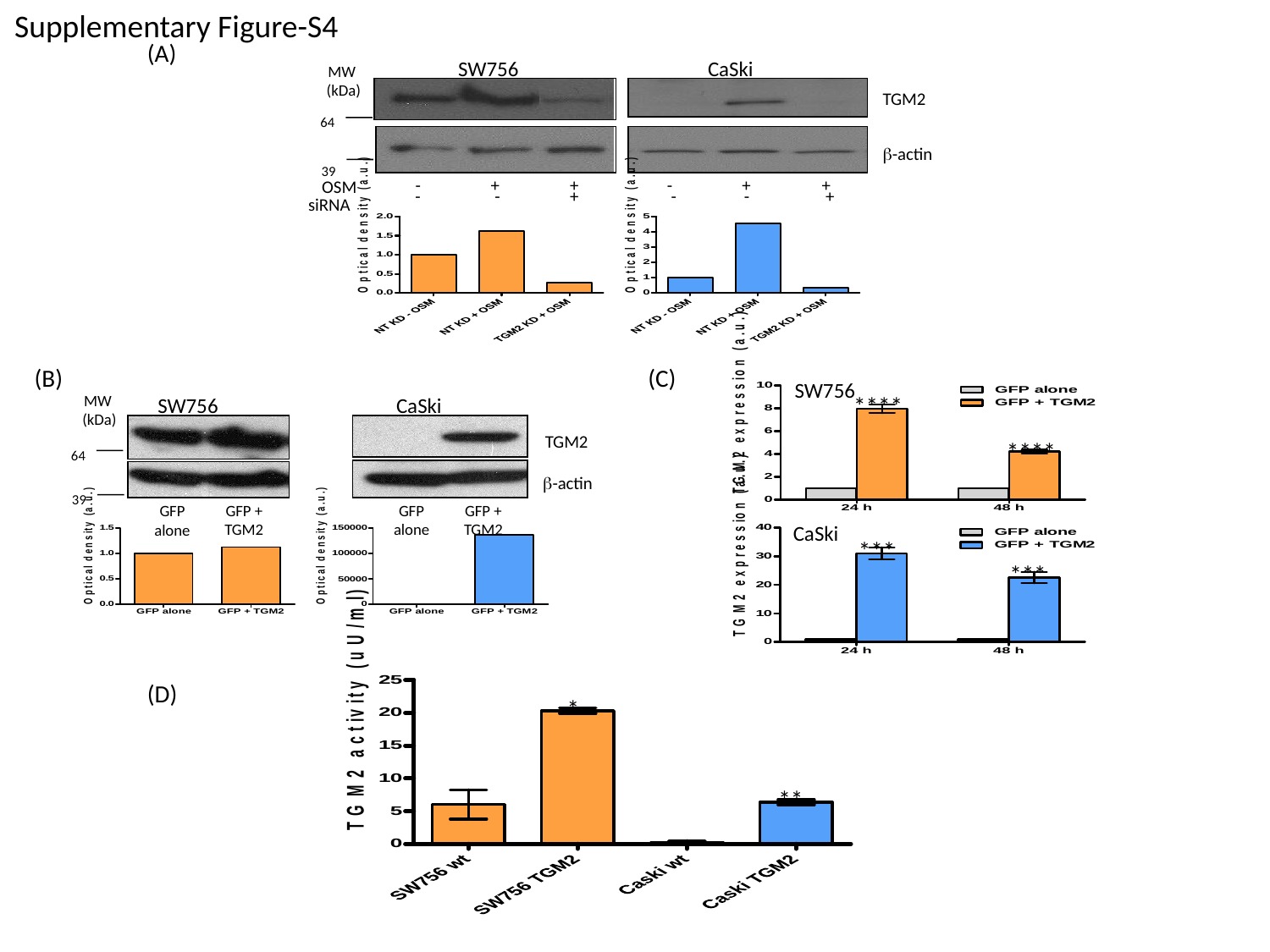

Supplementary Figure-S4
(A)
SW756
CaSki
MW
(kDa)
TGM2
64
b-actin
39
-
+
+
-
+
+
OSM
-
-
+
-
-
+
siRNA
(B)
(C)
SW756
****
****
MW
(kDa)
SW756
CaSki
TGM2
64
b-actin
39
GFP +
TGM2
GFP
alone
GFP +
TGM2
GFP
alone
CaSki
***
***
(D)
*
**
