# Supplementary material for: Tissue transglutaminase mediates the pro-malignant effects of oncostatin M receptor over-expression in cervical squamous cell carcinoma
Source: J Pathol. 2013 Sep 10;231(2):168–79. doi: 10.1002/path.4222 (PMC4288975; doi:10.1002/path.4222)
Supplement: Supplementary file 8 — Table S1. Power calculations for cell migration experiments. n, number of test cells; α, the type I error probability; m, ratio of control to experimental cells; δ, difference in population means; σ, within-group standard deviation; NT, non-targeting; KD, knockdown. As background levels of TGM2 were barely detectable in CaSki (see FigureA), TGM2 depletion was only performed in OSM-treated CaSki cells. [file path0231-0168-sd8.pptx]

## Slide 1
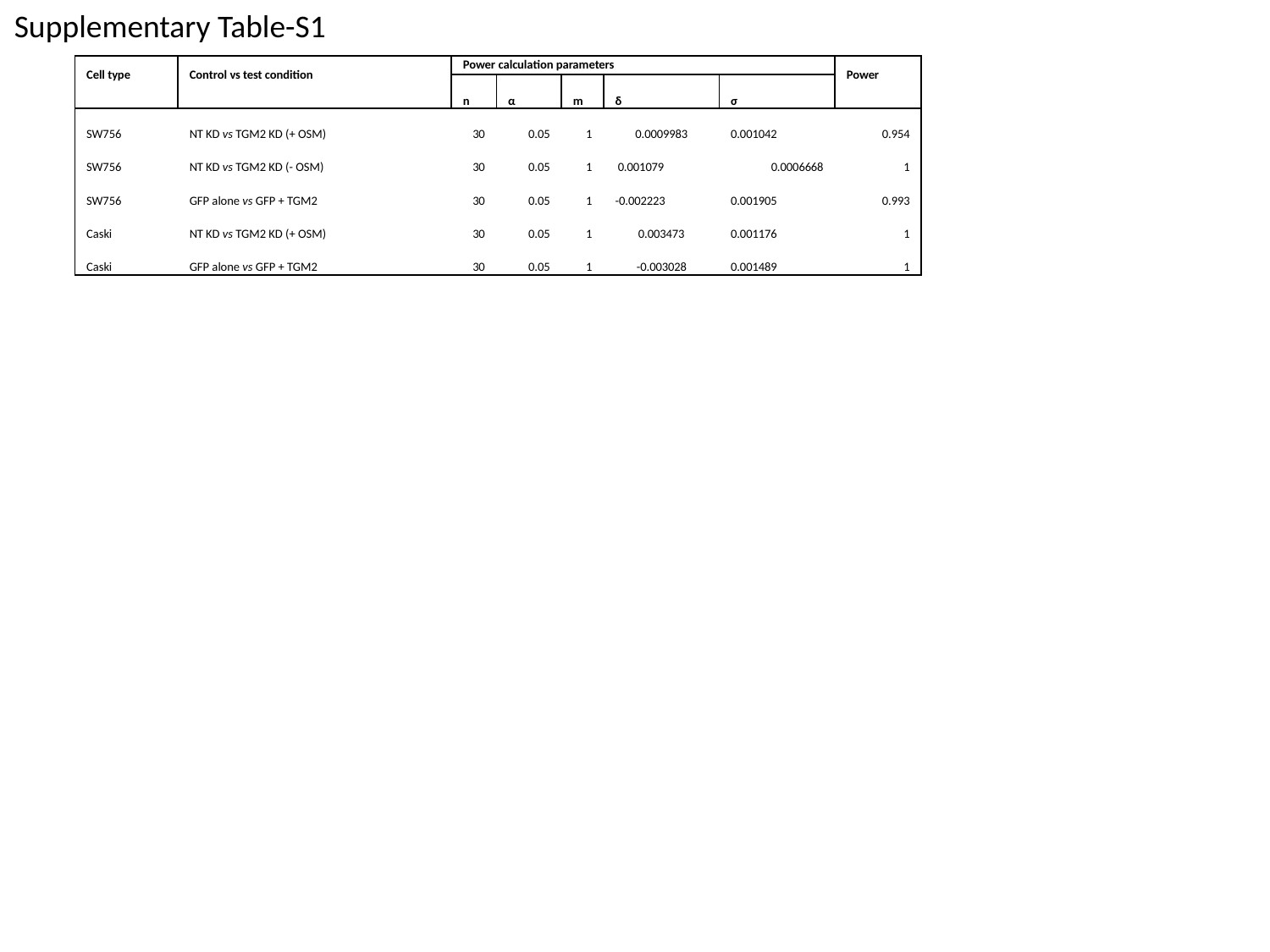

Supplementary Table-S1
| Cell type | Control vs test condition | Power calculation parameters | | | | | Power |
| --- | --- | --- | --- | --- | --- | --- | --- |
| | | n | α | m | δ | σ | |
| SW756 | NT KD vs TGM2 KD (+ OSM) | 30 | 0.05 | 1 | 0.0009983 | 0.001042 | 0.954 |
| SW756 | NT KD vs TGM2 KD (- OSM) | 30 | 0.05 | 1 | 0.001079 | 0.0006668 | 1 |
| SW756 | GFP alone vs GFP + TGM2 | 30 | 0.05 | 1 | -0.002223 | 0.001905 | 0.993 |
| Caski | NT KD vs TGM2 KD (+ OSM) | 30 | 0.05 | 1 | 0.003473 | 0.001176 | 1 |
| Caski | GFP alone vs GFP + TGM2 | 30 | 0.05 | 1 | -0.003028 | 0.001489 | 1 |
