# Supplementary material for: Tissue transglutaminase mediates the pro-malignant effects of oncostatin M receptor over-expression in cervical squamous cell carcinoma
Source: J Pathol. 2013 Sep 10;231(2):168–79. doi: 10.1002/path.4222 (PMC4288975; doi:10.1002/path.4222)
Supplement: Supplementary file 9 — Table S2. Primers used for real-time quantitative PCR. NA, not applicable. [file path0231-0168-sd9.pptx]

## Slide 1
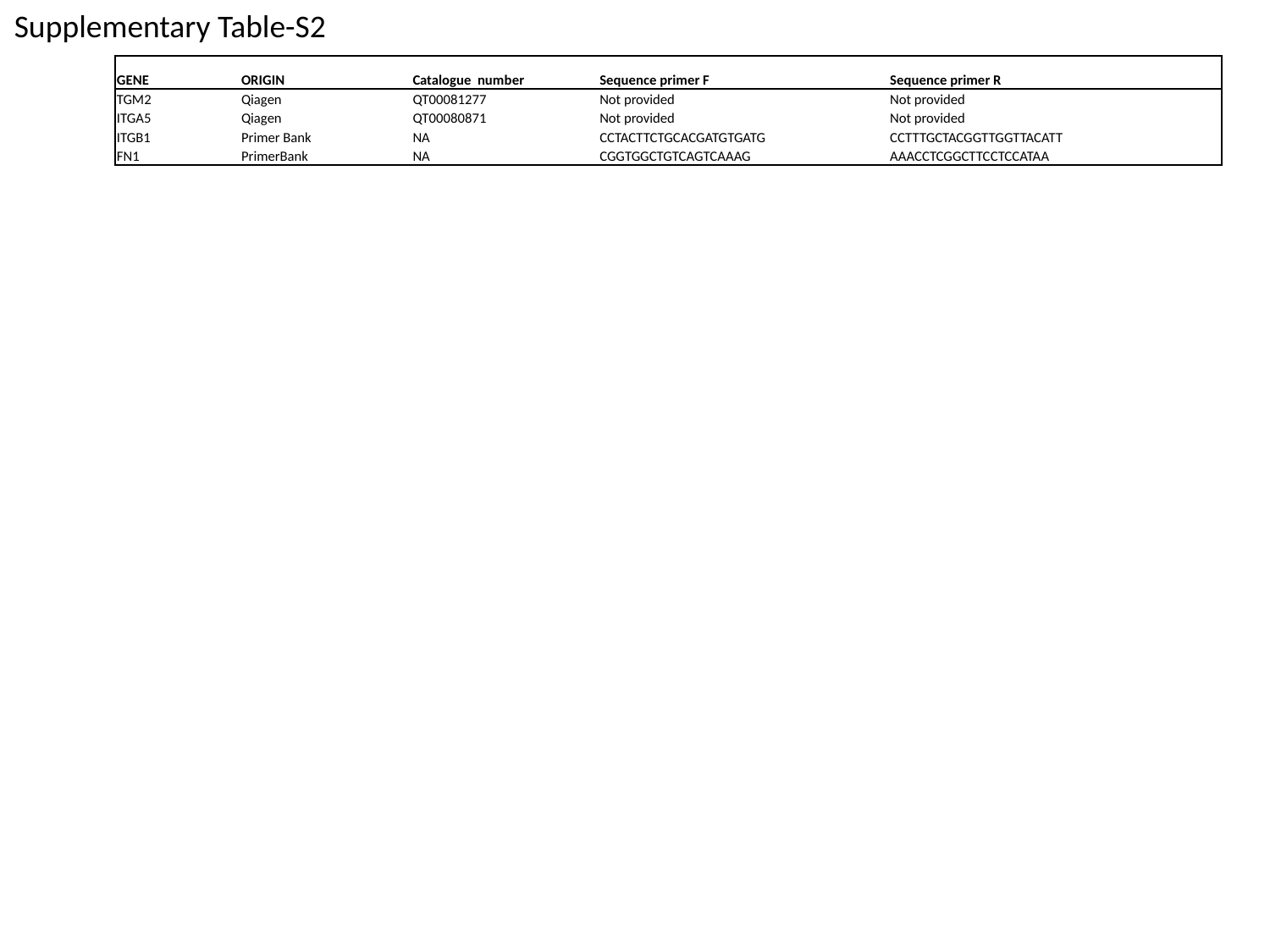

Supplementary Table-S2
| GENE | ORIGIN | Catalogue number | Sequence primer F | Sequence primer R |
| --- | --- | --- | --- | --- |
| TGM2 | Qiagen | QT00081277 | Not provided | Not provided |
| ITGA5 | Qiagen | QT00080871 | Not provided | Not provided |
| ITGB1 | Primer Bank | NA | CCTACTTCTGCACGATGTGATG | CCTTTGCTACGGTTGGTTACATT |
| FN1 | PrimerBank | NA | CGGTGGCTGTCAGTCAAAG | AAACCTCGGCTTCCTCCATAA |
