# Supplementary material for: Tissue transglutaminase mediates the pro-malignant effects of oncostatin M receptor over-expression in cervical squamous cell carcinoma
Source: J Pathol. 2013 Sep 10;231(2):168–79. doi: 10.1002/path.4222 (PMC4288975; doi:10.1002/path.4222)
Supplement: Supplementary file 10 — Table S3. Differential expression of OSMR–TGM2–integrin–α5β1–fibronectin pathway genes in cervical SCCs, according to OSMR levels. For each gene, the log2 ratio refers to expression levels in cervical SCCs with OSMR over-expression, compared with those without over-expression. The data were derived from our previous microarray profiling of sample set 1. Probe sets are ranked by p value. [file path0231-0168-sd10.pptx]

## Slide 1
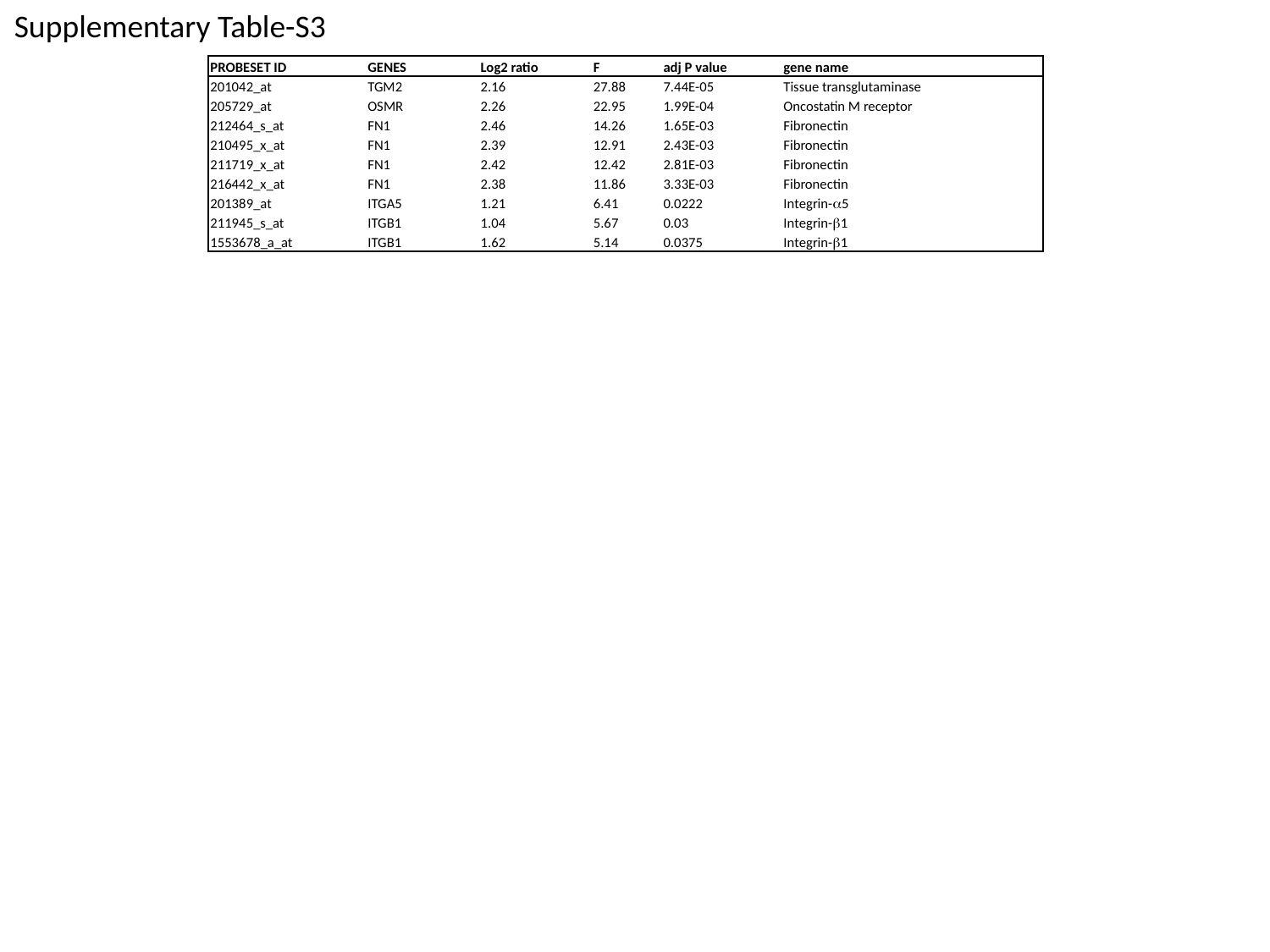

Supplementary Table-S3
| PROBESET ID | GENES | Log2 ratio | F | adj P value | gene name |
| --- | --- | --- | --- | --- | --- |
| 201042\_at | TGM2 | 2.16 | 27.88 | 7.44E-05 | Tissue transglutaminase |
| 205729\_at | OSMR | 2.26 | 22.95 | 1.99E-04 | Oncostatin M receptor |
| 212464\_s\_at | FN1 | 2.46 | 14.26 | 1.65E-03 | Fibronectin |
| 210495\_x\_at | FN1 | 2.39 | 12.91 | 2.43E-03 | Fibronectin |
| 211719\_x\_at | FN1 | 2.42 | 12.42 | 2.81E-03 | Fibronectin |
| 216442\_x\_at | FN1 | 2.38 | 11.86 | 3.33E-03 | Fibronectin |
| 201389\_at | ITGA5 | 1.21 | 6.41 | 0.0222 | Integrin-a5 |
| 211945\_s\_at | ITGB1 | 1.04 | 5.67 | 0.03 | Integrin-b1 |
| 1553678\_a\_at | ITGB1 | 1.62 | 5.14 | 0.0375 | Integrin-b1 |
